# Level of Heavy Metals and Potential Ecological Risks in Irrigated Horticultural Farms in the Vicinity of Lake Ziway, Central Ethiopian Rift Valley Region

**DOI:** 10.1155/2024/4724097

**Published:** 2024-05-14

**Authors:** GirmaTilahun Yimer

**Affiliations:** ^1^Hawassa University's Center for Ethiopian Rift Valley Studies (CERVaS), Hawassa, Ethiopia; ^2^Hawassa University, Department of Aquatic Sciences, Fisheries and Aquaculture, Hawassa, Ethiopia

## Abstract

Research on heavy metal pollution in horticultural farms located around lakes in the Central Ethiopian Rift Valley Region has focused on measuring the levels of heavy metals and their health implications. However, the ecological risks of horticultural farms contaminated with heavy metals in this region have not been studied. The current study addresses this gap by providing information on the degree of heavy metal contamination and the ecological risk associated with horticultural farms around Lake Ziway, using various pollution indices. An inductively coupled plasma optical emission spectrometer (ICP-OES) was used to measure the concentrations of nine heavy metals (As, Cd, Co, Cu, Cr, Ni, Pb, Hg, and Zn) in a total of 30 composite soil and irrigation water samples, each consisting of a mix of six subsamples. The results indicated that the mean concentrations of Hg, Cd, Pb, and Zn in soils collected from all the sampling sites exceeded the FAO/WHO maximum permissible limit (MPL). The values of both the contamination factor (CF) and contamination degree (Cd) of the heavy metals ranged from 0.04 to 2.66 and 2.81 to 6.14, respectively, indicating a low to medium level of contamination for both indices. The pollution load index (PLI) values of 0.451, 0.449, and 0.157 for sites 2, 1, and 3, respectively, indicate “unpolluted” to “moderately polluted” levels of heavy metal pollution. However, the ecological risk indices (ERIs) at sites 2 and 1 (158.92 and 141, respectively) showed a potentially high ecological risk due to soil pollution. Therefore, close monitoring and early intervention mechanisms must be in place to control pollution in the study area.

## 1. Introduction

The contamination of agricultural soil with heavy metals is a global environmental issue. This problem is primarily caused by the excessive and unregulated use of agrochemicals [1, 2] such as phosphate fertilizers, fungicides, herbicides, and insecticides that contain heavy metal impurities including As, Cd, Co, Cu, Hg, Ni, Pb, and Zn [3, 4]. Application of organic fertilizers such as livestock manure and compost has been reported as source of heavy metals in agricultural soils. These organic fertilizers contain high concentrations of heavy metals such as Cu, Zn, Cd, Ni, As, Pb, and Hg as contaminants [5].

Additionally, industrial effluent and urban wastewater are significant sources of different heavy metals that contaminate the nearby farmlands. It is well known that heavy metals are recalcitrant to degradation and can accumulate in the soil for extended periods if not taken up by plants or removed by leaching [6, 7]. Furthermore, their presence in the soil can lead to the accumulation and reach the harmful level in vegetables and crops grown in such contaminated soil, creating a health hazard for humans who consume these agricultural products [8].

In Ethiopia's Central Rift Valley region (CERVR), especially around Lake Koka and Lake Ziway, agrochemicals have been extensively used in irrigated horticultural farms in recent years. According to a survey by the Irrigation Development Authority Office of Ziway and Meki districts in CERVR, during the 2013/2014 crop season alone, 13,889 smallholder vegetable growers sprayed 53,044 L of pesticides and 50,057 kg of fungicide [9].

With regard to fertilizers, onion and tomato growers in the horticultural farms of CERVR use large amounts of urea and DAP fertilizers in three splits application, namely during transplanting, first and second cultivation and overturn of fertilizers applied to the surface layer of soil. For instance, a survey by Etissa et al. [10] found that 54.4% of onion growers apply an average of 230.35 kg·ha^−1^ DAP during transplanting, while 46.5% apply on an average of 188.29 kg·ha^−1^ DAP during the second split application and 17.8% apply an average of 119.4 kg·ha^−1^ DAP kg·ha^−1^ DAP at the last third split application.

Some studies have been conducted on the ecological risks associated with pesticides in Lake Ziway [11, 12]. Few studies have also been carried out on the levels and the health implications of heavy metal pollution in vegetables and fruits grown in irrigated farmlands around the lake [13, 14]. However, so far, no studies have been conducted to assess the potential ecological or environmental risks associated with heavy metals in agricultural soils around Lake Ziway. Therefore, this study aimed to fill the gap by providing information on the degree of heavy metal contamination and the ecological risk associated with horticultural farms around Lake Ziway, using various pollution indices.

Given the importance of Lake Ziway and its surrounding agricultural lands, it is critical to identify possible sources and investigate the potential hazards posed on the environment by heavy metals in the soil. Such study could help to inform decision makers and practices aimed at mitigating the risks associated with heavy metals in agricultural soils and promoting sustainable agriculture around Lake Ziway.

## 2. Materials and Methods

### 2.1. Description of the Study Area

The study area is located near Lake Ziway, approximately 140 km south of Addis Ababa. Lake Ziway is the largest freshwater lake in CERVR, with a surface area of 434 km^2^ and a maximum depth of 8.9 m, while also being the shallowest. The lake is home to several hippopotamuses, commercially valuable fish species, and indigenous bird species that nest on the five islands and the lake's shoreline. Recent study by Desta et al. [15] indicated that there have been major land use and land cover changes in the Lake Ziway region. According to these authors, agricultural and settlement areas have expanded from 57% in 1973 to 75% in 2014, resulting in deforestation, which has reduced the forest cover from 26.16% to 6.63%.

**Figure 1:**
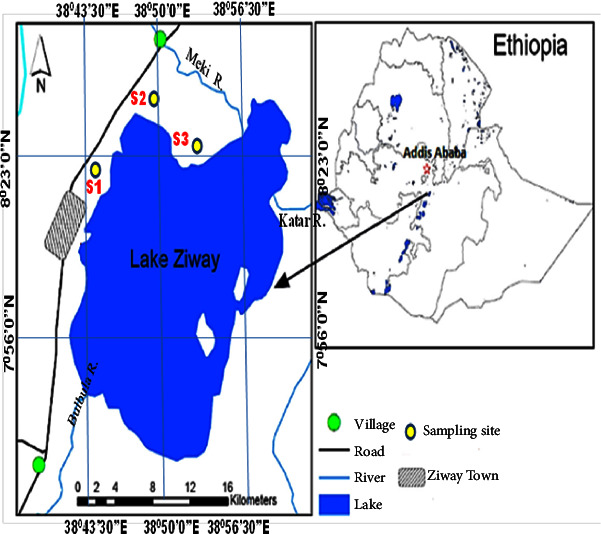
Lake Ziway and location of the three sampling sites.

The horticultural farm in the study area is located between Meki and Ziway towns and borders Lake Ziway and the international highway to Kenya (Figure 1). The highway is a heavily trafficked two-way road where commercial trucks, buses, and other vehicles frequently park at the roadside very near to the farmlands for maintenance, lubrication, and oiling. This a potential source to contaminate the agricultural soil through water runoff. Spanning an estimated length of 15 km and a width of 1 to 1.5 km, the farm is primarily dominated by smallholder horticultural farmers that use agrochemicals, including phosphate and nitrogen fertilizers (DAP and urea), and pesticides, primarily organophosphates and organochlorines throughout the year without discrimination.

The horticultural farm also serves as a corridor for numerous livestock that travel back and forth between the lake and the neighbouring dry lands for water daily, for most of the year. Despite the potential for heavy metal pollution arising from impurities of agrochemicals and contaminants from livestock manure and vehicle emissions, the study area is well known for year-round production of a wide variety of vegetables, including tomatoes and onions, which are distributed to local markets and Addis Ababa, Ethiopia's capital city.

### 2.2. Sampling Site Selection

Three sampling sites were purposely selected as hotspots between Lake Ziway and the Ethiopia-Kenya highway. These sites are S1 (Abunea-Germama), S2 (Wellibula), and S3 (Bekelae Girrissa), each with its local name in brackets. Horticultural farms, mainly growing tomatoes and onions, are densely clustered in these sites and are irrigated by small-scale irrigation systems throughout the year. Farmers in these areas also use agrochemicals such as fertilizers and pesticides in large quantities to increase productivity and maximize profits per unit area.

### 2.3. Sample Collection and Pretreatment

#### 2.3.1. Soil Samples

Fifteen composite soil samples at 0–20 cm depth were taken from randomly selected tomato and onion seedling beds/plots from three sampling sites using a stainless steel auger. This was done in August 2022 (during the rainy season), following the procedure reported by Mondal [16]. Each soil sample weighed about 1 kg, obtained by collecting six subsamples in a zig-zag pattern and pooled together to form a composite sample. The soil samples were then carefully packed into clean polyethylene bags, labelled, and immediately transported to HORTICOOP ETHIOPIA, the agricultural laboratory centre in Debre Zeit which is approximately 80 km from the sampling site. In the laboratory, the soil samples were first air-dried in a dust-free area at room temperature (22 to 25°C) for five days. After that, they were oven-dried until a constant weight was achieved. The dried samples were then ground with a mortar and pestle until they passed through a 2 mm sieve and were homogenized. Finally, the homogenized soil samples were carefully stored in clean polyethylene bags and kept in desiccators until digestion and heavy metal analysis.

#### 2.3.2. Irrigation Water Samples

Water for irrigation was diverted from Lake Ziway through an open surface channel to reach farm plots. Water samples entering the farm plot were collected using a precleaned, dry, high-density polyethylene bottle rinsed with the sample water. 500 ml of water from each farm plot, with a total of 15 samples (five from each sampling site), was collected. The samples were immediately placed in an ice box and transported to the HORTICOOP laboratory at Debre Zeit for heavy metal analysis.

### 2.4. Sample Digestion and Heavy Metal Analysis

Acid digestion was performed on soil and irrigation water samples to determine the presence of nine heavy metals (As, Cd, Co, Cu, Cr, Ni, Pb, Hg, and Zn) using inductively coupled plasma optical emission spectrophotometry (ICP-OES). For the soil sample, a mixture of 8 ml of a 3 : 1 HNO_3_ : HCl solution was added to 1.25 g of air-dried ground soil in a borosilicate digestion flask. The mixture was heated to 200°C for one hour and then cooled and filtered. The filtrate was transferred to a 50 ml volumetric flask and diluted to 25 ml with deionized water, and a blank digest was carried out similarly. Likewise, a 50 ml aliquot mixed with 1 ml of conc. HNO_3_ was heated on a hot plate at 95°C until the volume was reduced to 15 ml. The sample was then cooled and filtered using Whatman filter paper No. 42, and a blank digest was carried out similarly. Both digests were used to determine the presence of nine heavy metals.

### 2.5. Limit of Detection (LOD) and Limit of Quantification (LOQ)

The limit of detection (LOD) was determined by calculating three times the standard deviation of the replicate analysis of the blanks. Likewise, the limit of quantification (LOQ) was determined by calculating ten times the standard deviation of the blank prepared by the optimized procedure for each heavy metal. The summarized results are provided in Table 1. Accordingly, the LOD for targeted heavy metals in water ranged from 0.002 to 0.137 mg/L, while the corresponding LOQ range was 0.006 to 0.458 mg/L. The LOD for heavy metals in soil ranged from 0.035 to 0.290 mg/kg, while the corresponding LOQ range was 0.118 to 0.967 mg/kg.

### 2.6. Method Validation

The accuracy of the analytical method was confirmed by conducting a recovery study using the spiking experiment. The results showed that the recovery percentage ranged from 100.2% to 118.1% for soil samples and 97.3% to 117.3% for water samples (as shown in Table 2), which is within the acceptable range. Additionally, the % RSD for almost all measured values for both water and soil samples was lower than 10%.

### 2.7. Assessment of Level of Contamination and Ecological Risk

The following indices were used to assess the contamination level and potential ecological risk of heavy metals in the soil samples collected from irrigated horticultural farms near Lake Ziway: Contamination factor (CF), Degree of total environmental contamination (Cd), Pollution Load Index (PLI), Single Element Potential Risk Factor (ER), and Potential Ecological Risk Index (ERI). Each index has a brief description and equation for calculation.

#### 2.7.1. Contamination Factor (CF)

CF is an index applied to assess the pollution level of a single heavy metal and shows site-specific soil contamination by the targeted heavy metal. CF is calculated using the following equation [17, 18]:

(1) $$\begin{array}{c} \text{CF}=\frac{C_{s}}{C_{r}}, \end{array}$$

where *C*_*s*_ = the mean metal concentration of the specific metal at the sampling site and *C*_*r*_ = concentration of a given element in the reference/baseline or background. There were no data on the heavy metal concentrations at the study site or the national level in Ethiopia. Instead, the concentrations of elements in urban soils listed in Alekseenko and Alekseenko [19] were used as reference. These authors have compiled the average concentrations of elements in urban soil from more than 15 years of special studies conducted in over 300 cities and settlements across Europe, Asia, Africa, Australia, and America.

Since the study area is peri-urban and located between rapidly growing neighbouring towns, the following reference values for the heavy metal concentrations (in mg·kg^−1^) were used in all sampling sites: 15.9 (As), 0.9 (Cd), 14.1 (Co), 80 (Cr), 39 (Cu), 0.88 (Hg), 33 (Ni), 54.5 (Pb), and 158 (Zn).

CF was evaluated using the four-level categories of contamination: low (CF < 1), moderate (1 ≤ CF < 3), considerable (3 ≤ CF < 6), and very high (CF ≥ 6), indicated by Hákanson [20].

#### 2.7.2. Degree of the Total Environmental Contamination (*C*_*d*_)

*C*_*d*_ is the total CF of all metals detected and measures the degree of general contamination at the specific sampling site. It is an index of multielement calculated using the following equation [21]:

(2) $$\begin{array}{c} C_{d}=\overset{i=1}{\underset{n}{\sum }}CF, \end{array}$$

where *n* = number of analysed metals, *i* = *i*th pollutant metal, and CF = contamination factor. *C*_*d*_ is interpreted as follows: *C*_*d*_ < 6 = low; 6 ≤ *C*_*d*_ < 12 = moderate; 12 ≤ *C*_*d*_< 24 = considerable; and *C*_*d*_ ≥ 24 = very high (severe anthropogenic pollution), classified by Hákanson [20] to show a degree of contamination.

#### 2.7.3. Pollution Load Index (PLI)

The PLI for the entire sampling site can be determined as the nth root of the product of the CFn as indicated in the following equation:

(3) $$\begin{array}{c} \text{PLI}=\left(\text{CF}1\;X\text{ CF}2\;X\text{ CF}3\;X\cdots \text{XCFn}\right)\;\frac{1}{n}. \end{array}$$

In this equation, CF is the contamination factor obtained from the contamination factors for each metal, while *n* is the number of heavy metals analysed. The calculated PLI values were interpreted as follows.

0 = background concentration, 0 > PLI ≤ 1 = unpolluted to moderately polluted, 1 > PLI ≤ 2 = moderately polluted, 2 > PLI ≤ 3 = moderately to highly polluted, 3 >PLI ≤ 4 = highly polluted, and PLI >4 = very highly polluted [22–24].

#### 2.7.4. Single Element Potential Ecological Risk Factor (ER)

Er, which expresses the potential ecological risk of a single heavy metal risk, is determined based on the metal's contamination factor (CF) and its toxic response coefficient (TR). The TR values for each targeted heavy metal are as follows: As = 10, Pb = 5, Zn = 1, Cd = 30, Hg = 40, Cu = 5, Ni = 5, Co = 5, and Cr = 2 [20, 25, 26].

In this study, equation (4) was used to determine the ecological risk (ER) of the soils of the horticultural farms at the study site.

(4) $$\begin{array}{c} \text{ER}=\text{TR XCF}, \end{array}$$

where CF is the pollution factor, ER indicates the ecological risk of each targeted heavy metal, and TR indicates the toxicity of the targeted heavy metal. A single heavy metal's potential ecological risk (ER) degree is interpreted as follows.

ER < 40 = low, 40 < ERI < 80 = moderate, 80 < ERI < 160 = considerable, 160 < ERI < 320 = high, and ERI >320 = very high [20].

#### 2.7.5. Potential Ecological Risk Index (ERI)

The ERI is the total of a specific sampling site's single-element ecological risk factors (ER). It emphasizes the toxicology of heavy metals or overall heavy metal pollution and evaluates the potential ecological risk. It is calculated using the following equation:

(5) $$\begin{array}{c} \text{ERI}=\overset{i=1}{\underset{n}{\sum }}\text{ER}. \end{array}$$

The degree of ecological risk to the environment expressed in ERI values is ranked as follows.

ERI < 100 = low, 100 < ERI < 150 = moderate, 150 < ERI <200 = considerable, 200 < ERI < 300 = high, and ERI > 300 = very high [20].

## 3. Results and Discussion

### 3.1. Level of Heavy Metals in Agricultural Soils

Table 3 displays the levels of heavy metals found in soil samples collected from the three distinct agricultural sites, with an intention to detect patterns and enable comparisons. The mean concentration of heavy metals across all sites followed the order Zn > Pb > Cr > Cu > Ni > As > Co > Hg > Cd. Statistical analysis revealed significant differences in the mean concentration of heavy metals among the three sampling sites (*p* > 0.05). Specifically, the soil collected from site 2 had the highest levels of six heavy metals, namely, Pb, As, Ni, Co, Hg, and Cd. Conversely, the highest values of the remaining three heavy metals, namely, Zn, Cr, and Cu, were observed in the soil samples collected from site 1.

In contrast, the soil samples collected from site 3 showed the lowest concentrations of all nine heavy metals. This indicated that the agricultural soil at this site was comparatively less contaminated than the other two sites. The lower levels of contamination can be attributed to the site's limited exposure to intensive farming and overzealous human activities, as well as its distance from the main road in comparison to sites 1 and 2. These results have significant implications for agricultural management practices and highlight the importance of mitigating human activities that may lead to soil contamination.

It is noteworthy that the levels of five heavy metals, namely, As, Cu, Ni, Co, and Cr, found in soil samples from all study sites, were below the maximum permissible levels (MPLs) for agricultural soil, as stipulated by the by FAO/WHO in 1993 and 2002 guidelines. On the other hand, the soil contamination and ecological health in the study area were severely affected by four heavy metals, namely, Hg, Cd, Pb, and Zn.

The mean concentrations of Hg (1.96 mg/kg) and Cd (1.0 mg/kg) detected in soil samples from all the sites have considerably exceeded the recommended MPL by FAO/WHO, which is 0.3 mg/kg for both elements. Similarly, the average concentrations of Pb (30.9 mg/kg) and Zn (56.4 mg/kg) were higher than the FAO/WHO's maximum permissible limit of 10 mg/kg and 50 mg/kg, respectively.

The high levels of the aforementioned four heavy metals can be attributed to the excessive use of metal-based pesticides and fertilizers. This is in line with related investigations that have linked the prolonged application of pesticides and fertilizers with their accumulation in agricultural soils [29, 30]. Similar studies have also reported that the continuous use of herbicides, insecticides, fungicides [31], and fertilizers [32] can contribute significantly to the accumulation of heavy metals in agricultural soils.

Phosphate rocks, their fertilizer blends, and micronutrient supplements may also be contaminated with Cd, Pb, and Zn [4, 33] which may add to their enrichment in receiving soils. It has also been documented that the Pb-Zn-Cu-Cd-Hg group could be derived from anthropogenic sources and atmospheric deposition, which could be considered a significant source of contaminants in both peri-urban and agricultural soils [4, 34].

The explanation provided appears to be plausible account of potential soil contamination in the peri-urban horticultural farms in the study areas, located between the fast growing towns of Meki and Ziway. The area is dominated by smallholder horticultural farmers who employ pesticides, including organochlorines and organophosphates [35], and fertilizers, such as urea, DAP, and NPK blends [10], without discrimination.

The problem of soils contamination by heavy metals in the study area is further exacerbated by the illegal trade of unregistered, unlabelled, and repackaged pesticides and fertilizers in shops and open markets in nearby towns, such as Koka, Meki, Ziway, and Adamitulu [36]. Furthermore, the study area is predominantly exposed to diffused sources of pollutants that can infiltrate the irrigated horticultural farms through surface runoff and atmospheric deposition from the burning of charcoal, vehicles, and effluents from neighbouring floriculture and peri-urban areas [37]. Additionally, the study sites, particularly Site 1 and Site 2, are subject to overzealous human activities and are in close proximity to the busy Ethiopia-Kenya highway, thus magnifying the possibility of heavy metal pollution.

The mean concentration of mercury (Hg) in the soil of all sampling sites near Lake Ziway, which is 1.96 mg/kg, as revealed by this study is a cause for concern as it exceeds the maximum permissible limit set by FAO/WHO [27, 28] by more than six times. The recorded mean concentration falls between 0.58 and 1.8 mg/kg, which is within the range of background contents of Hg in soils worldwide, according to Nance et al. [38]. Recent studies, including the findings of Xu et al. [39], have also indicated that the concentration of Hg in soils has significantly increased by a factor of 3 to 10, which is in line with the present study. The authors attributed the upsurge of Hg to the combustion of fossil fuels or charcoal burning and long-range atmospheric transport processes.

It is important to note that elevated levels of Hg in soils pose a high ecological risk and can have negative implications for human health. Most forms of Hg are highly toxic to humans, and even low levels of exposure can seriously affect the central nervous system, as documented by Clarkson [40] and Nance et al. [38]. Additionally, Hg is known for its biomagnification and bioconcentration and is slowly metabolized, as noted by Kid et al. [41] and Rice et al. [42].

**Figure 2:**
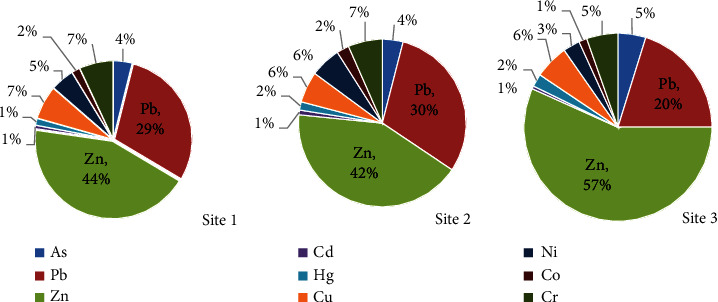
Percentage contribution of each heavy metal to the total load in agricultural soils collected from the three sampling sites.

The data presented in Figure 2 show that Zn and Pb are the two heavy metals that contributed the most, 46.3% and 25.4%, respectively, to the overall heavy load in agricultural soils collected from all sampling sites. The percentage contribution of these heavy metals to total heavy metal load in soil samples collected from each sampling site ranged from 42.2% to 57.0% for Zn and 20.0% to 30.4% for Pb. Although Zn is an essential micronutrient that actively participates in plant metabolic and physiological processes, promoting plant growth hormones and proteins [43], it can be toxic to soil microorganisms that improve soil fertility and structure [44].

Similarly, Pb has been classified as a hazardous heavy metal pollutant because of its high toxicity [45]. Prolonged exposure even to low concentrations of Pb leads to high toxic levels [44]. Apart from reducing soil nutrients, microbial diversity, and soil fertility [46], the transfer of Pb from soil and its accumulation in plants can cause DNA damage, reduction of chlorophyll content, and inhibition of seed germination [44].

As depicted in Table 4, the findings of the current study are compared with those of other researchers in other horticultural farms located in the Central Ethiopian Rift Valley. Accordingly, the recorded values for all eight heavy metals were much higher in Modjo and Koka vegetable farms, except for Pb, as reported by Gebeyehu and Bayissa [48] and Bayissa and Gebeyehu [49]. However, Samuel et al. [47] found that soils collected from vegetable farms in the Hawassa industrial zone had more or less equal values for As, lower values for Pb and Cd, and considerably higher values for Zn, Cu, Ni, and Cr than the current study.

The difference in the concentrations of heavy metals in soils collected from the four study areas can be attributed to the source of pollutants. For instance, considerably high concentrations of Cr ranging from 35.93 to 60.73 mg·kg^−1^ were found in Modjo and Koka vegetable farms that received river water contaminated with effluents from leather factories. Likewise, soils collected from the Hawassa vegetable farm irrigated by river water receiving effluent from textile factories had mean Cr concentrations as high as 26.1 mg·kg^−1^, which is still much higher than concentrations of Cr recorded in Ziway farm.The reason behind the much higher Cr values in the three vegetable farms is because Cr is the primary pollutant discharged from textile and leather factories near these three study areas. As opposed to these three vegetable farms, Ziway farms had much lower mean concentrations of Cr, measuring as low as 3.4 mg·kg^−1^, and the contaminants mainly originate from agrochemicals such as pesticides and fertilizers that are diluted by lake water.

### 3.2. Levels of Heavy Metals in Irrigation Water

The results presented in Table 5 indicate that the concentration of heavy metals in irrigation water exhibited a specific order: Pb > Ni > Zn > As > Cu > Hg > Cr > Co = Cd. The maximum values of four heavy metals, namely, Pb, Zn, Cu, and As, were detected in the irrigation water collected from site one. On the other hand, five heavy metals (Ni, Hg, Cr, Co, and Cd) had their maximum values in irrigation water collected solely from site two. Conversely, the lowest concentrations of six heavy metals (Pb, Zn, As, Cu, Co, and Cd) had their minimum values in irrigation water samples collected from site three, a similar status as evidenced by the results obtained from the soil samples. This suggests that both the agricultural soil and the irrigation water at this site are contaminated by heavy metals from similar sources, with a difference in that the latter is diluted while the contaminants accumulate in the soil.

The concentrations of heavy metals in all irrigation water samples were found to be within the maximum limit recommended by the FAO [50] for vegetable farms. This could be due to the use of an irrigation method where pumped lake water flows in long furrows constructed up to the vegetable farms, causing dilution of the heavy metals. In a study conducted by Mekonen et al. [51], who examined the distribution of mercury (Hg) in sediments from various freshwater bodies, it was reported that the concentration of Hg in ten sampling sites of Lake Ziway ranged from 17 to 119 *μ*g·kg^−1^, with an average value of 44 *μ*g·kg^−1^. The authors attributed the higher concentrations of Hg in the sediments of Lake Ziway to the presence of small and large-scale horticultural farms near the lake.

In the present study, the lake water used for irrigation had a Hg content ranging from 60 to 110 *μ*g·kg^−1^, with an average value of 90 *μ*g·kg^−1^. These levels are higher than the previously reported Hg load in the lake sediment by Mekonnen et al. [51]. In contrast, Woldetsadik et al. [52] found lower levels of metals in irrigation water collected from ten sampling sites of Addis Ababa vegetable farms. These authors reported mean concentrations (*μ*g·L^−1^) ranging from 0.17 to 2.12 for Cd, 5.97 to 36.5 for Ni, 9.48 to 47.7 for Pb, 14.2 to 35.1 for Cr, and 11.2 to 88.4 for Zn. The authors attributed these low values for the heavy metal to the minimal industrial activities in the region and the dilution of wastewater with stream or river water.

### 3.3. Levels of Contamination and Ecological Risk

#### 3.3.1. Contamination Factor (CF), Degree of Contamination (Cd), and Pollution Load Index (PLI)

Table 6 displays the site-specific contamination of a single heavy metal, which is the contamination factor (CF), with values ranging from 0.04 to 2.66 for the three sampling sites. These values generally followed the order of Hg > Cd > Pb > Zn > As > Co > Cu > Ni > Cr across the three sampling sites. Since CF values indicate the soil contamination by each heavy metal [20], the values obtained for Ziway horticultural farm fall in the ranges of low to moderate contamination.

The total sum of CF values for all metals detected at specific sampling sites, known as *C*_*d*_, varied from 2.81 to 6.14 and followed the order: Site 2 > Site 1 > Site 3. The *C*_*d*_ values suggest a low to moderate level of soil contamination by heavy metals for the three sampling sites, according to Hákanson's classification (1980). The four heavy metals (Hg, Cd, Pb, and Zn) contributed the most to the overall contamination (*C*_*d*_), with percentage values of 78.4%, 79.2%, and 84.5% at sites 1, 2, and 3, respectively. Mercury (Hg) alone contributed from 38.6% to 56.6%, indicating that soil contamination by Hg poses a high ecological risk in the study area.

The Pollution Load Index (PLI) allows to compare the levels of heavy metal pollution at the three different sampling sites. Accordingly, site 2 had the highest PLI value of 0.467, followed by site 1 with a value of 0.449, and site 3 with the lowest value of 0.157. As per the guidelines of Tomilson et al. [24]; Angulo [22]; and Ho and Aj [23], these values fall under the 0 < PLI < 1 category of “unpolluted to moderately polluted” levels of heavy metal pollution. It is worth noting that these calculated values are much lower than the values reported by Samuel et al. [47] who found PLI values of 2.95 and 3.69 for Hawassa vegetable farms near the Hawassa Textile Factory. These values indicate a highly polluted status falling in “the near and the 3 < PLI < 4 category,” respectively.

#### 3.3.2. Single Element Potential Ecological Risk Factor (ER) and Potential Ecological Risk Index (ERI)

Table 7 depicts the levels of ER (Enrichment Ratio) for a single heavy metal at three different sampling sites. Results showed that the values ranged from 0.08 to 106.4, with chromium (Cr) having the lowest level at site three and mercury (Hg) having the highest level at site two. Based on these data, it was found that the potential risk of each targeted heavy metal varied from low to considerable levels according to Hákanson's method (1980). The ER pattern for Site 1 and Site 2 followed a similar pattern, with Hg > Cd > Pb > As > Co > Cu > Ni > Zn > Cr. However, a slight difference in the ER pattern was observed at site 3, which was Hg > Cd > As > Pb > Cu > Co > Ni > Zn > Cr. Furthermore, Ecological Risk Index (ERI) for the three sampling sites followed the order: Site 2 > Site 1 > Site 3, with corresponding values of 158.92, 141.55, and 77.86, respectively. According to Hákanson's method (1980), the calculated ERI values at each sampling site revealed that the ecological risk from the targeted heavy metal pollution would be low, moderate, and considerable for site 3, site 1, and site 2, respectively.

### 3.4. Relationships, Distributions, and Sources of Soil Pollution

#### 3.4.1. Correlation between Heavy Metals in Soils

The present study investigated the correlation coefficients of heavy metals in the soils of Ziway irrigated horticultural farms.  Table 8 indicates that there are significant positive correlations (*p* < 0.01) between several heavy metals. Notably, there was a strong positive correlation between As and Ni (*r* = 0.96), Cd (*r* = 0.94), Zn (*r* = 0.92), Cr (*r* = 0.89), and Hg (*r* = 0.85). Similarly, Zn exhibited a strong correlation with Ni (*r* = 0.93), Cd (*r* = 0.88), Cr (*r* = 0.88), and Hg (*r* = 0.77). Cd was strongly correlated with Ni (*r* = 0.96), Cr (*r* = 0.95), and Hg (*r* = 0.71), while Pb was correlated with Co (*r* = 0.99) and Cu (*r* = 0.75). Additionally, there was a significant positive correlation between Hg and Ni (*r* = 0.80), Cu and Co (*r* = 0.75), and Ni and Cr (*r* = 0.91). Furthermore, there were significant positive correlations (*p* < 0.05) between Pb and Cr (*r* = 0.58), Pb and Cd (*r* = 0.55), Hg and Cr (*r* = 0.63), and Co and Cr (*r* = 0.54).

The observed high positive correlation among heavy metals indicated the existence of a common source of pollution. The most likely source of this pollution was the excessive use of fertilizers and pesticides in horticultural farms located in Ziway. This is in line with the works of Lv et al. [53] and Marrugo-Negrete et al. [54] in Eastern China and Colombia, respectively, who reported a common source of pollution in paired heavy metals that are significantly and positively correlated. Similarly, Samuel et al. [47] reported comparable findings, where significant positive correlations were observed between heavy metals in soils collected from industrial sites located in Hawassa. For instance, they found a positive correlation between As and Cd (*r* = 0.71), As and Cr (*r* = 0.59), Zn and Ni (*r* = 0.41), and Pb and Cu (*r* = 0.82).

#### 3.4.2. Principal Component Analysis (PCA)

The present study utilized principal component analysis (PCA) with the varimax rotation approach to ascertain the sources of nine targeted heavy metals. The results indicate that two principal components, PC1 and PC2, possessed eigenvalues greater than 1, thereby accounting for 90% of the overall variance in heavy metals in the soil. The first principal component (PC1) exhibited the highest percentage of variance (69.0%), encompassing heavy metals such as As, Ni, Cd, Hg, Zn, and Cr. This finding indicated that human activities, such as the excessive or indiscriminate use of synthetic fertilizers and pesticides, have contributed to the accumulation of these heavy metals in the soil. This finding is consistent with previous studies by Gupta et al. [55].

The analysis of the soil's second principal component (PC2) shows that it accounts for 21.0% of the total variance. This component is predominately influenced by Pb (0.949), Co (0.856), and Cu (0.808) as shown in Table 9, suggesting the presence of both natural and artificial contaminants in the soil. The artificial contaminants could have originated from vehicle emissions, as noted by Wang et al. [56] in China. This is plausible as the study area is peri-urban and the Ziway horticultural farms are situated along the main Ethiopia-Kenya highway.

**Figure 3:**
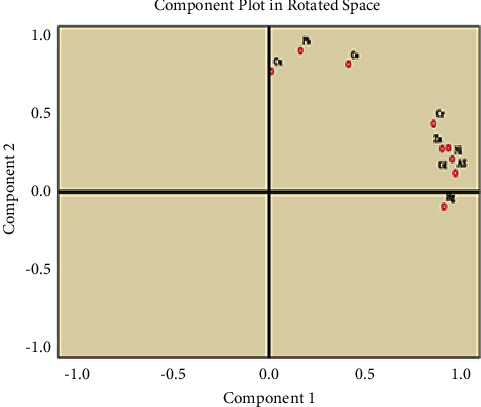
Plot of the first and second PC loading vectors of the nine targeted heavy metals in soils from Ziway horticultural farms.

The relationship between the heavy metals in the two principal components is illustrated in Figure 3. The plot shows that most heavy metals were clustered, distinguishing them from the other group. As a result, in PC1, six out of the nine targeted heavy metals (As, Cd, Ni, Zn, Cr, and Hg) were clustered on the right of the loading plot and were found to be positively correlated. This suggests that they have a common source of contamination. Similarly, PC2 was characterized by three heavy metals (Pb, Cu, and Co) that were clustered together in the loading plot, meaning that they are from the same source of contamination.

## 4. Conclusions

The objective of the present study was to evaluate the levels of heavy metals and the potential ecological risks in soil samples collected from various horticultural sites around Lake Ziway. The results revealed that all the soil samples contained nine targeted heavy metals. Four heavy metals, namely, Hg, Cd, Pb, and Zn, had mean concentrations above the permissible limit set by FAO/WHO. Based on the findings, it is crucial to take appropriate action to prevent and mitigate the negative impacts of these four heavy metals, particularly Hg, which exceeded the permissible limit by more than six times. Measures such as the implementation of sustainable agricultural practices, proper disposal of hazardous wastes, and the use of eco-friendly pesticides and fertilizers can help reduce the risk of soil pollution in the area.

Upon further data analysis, it was known that there are high potential ecological risks due to soil pollution at sites 1 and 2. This was evident from the environmental risk index (ERI) values of 158.92 and 141, respectively. These findings imply that the soil in these sites is contaminated with heavy metals beyond the recommended levels. The contamination is mainly attributed to the excessive and indiscriminate use of both registered and unregistered pesticides and fertilizers by smallholder horticultural farmers. Also, the proximity of the sites to the busy international road contributes to the pollution.

The results of the study provided valuable insights into the correlation patterns and principal component analysis (PCA) among heavy metals found in the soils of Ziway irrigated horticultural farms. The high positive correlation observed in PCA suggested the existence of two clusters. The first cluster, PC1, included six heavy metals (As, Ni, Cd, Hg, Zn, and Cr), while the second cluster, PC2, was influenced by three heavy metals (Pb, Cu, and Co). Each cluster suggested suspected source of pollution. However, to identify the source of contamination with certainty, further qualitative and quantitative studies are required.

In general, the present study underscores the importance of monitoring the presence and concentrations of heavy metals in soil used for irrigated horticultural farming in the vicinity of Lake Ziway. The research findings can serve as a benchmark for further investigations and facilitate the formulation of appropriate measures to address heavy metal pollution. Therefore, it is recommended that stakeholders, including horticultural farmers, the government, and other relevant bodies, work together to ensure the safety of horticultural products and the environment in irrigated horticultural farms in the vicinity of Lake Ziway to ensure the sustainability of agriculture in the neighbourhood of Lake Ziway.

## Figures and Tables

**Table 1:** LOD and LOQ for heavy metal analysis in water (mg/L) and soil (mg/kg) samples.

Parameter	Heavy metal								
	As	Cd	Co	Cu	Cr	Ni	Pb	Hg	Zn
LOD for water	0.03	0.002	0.006	0.017	0.027	0.062	0.075	0.017	0.046
LOQ for water	0.1	0.006	0.021	0.058	0.090	0.208	0.252	0.058	0.153
LOD for soil	0.211	0.035	0.113	0.290	0.119	0.177	0.099	0.116	0.131
LOQ for soil	0.702	0.118	0.378	0.967	0.398	0.590	0.329	0.388	0.437

**Table 2:** Percentage of recovery of the method used for soil and water samples analysed (mean ± SD, *n* = 3).

Heavy metals	Soil sample concentration (amount in mg/kg)			% recovery	% RSD	Water sample concentration (amount in mg/L)			% recovery	% RSD
	A	B	C			A	B	C		
As	0.105 + 0.004	1	1.248 + 0.002	114.3	4.15	0.16 + 0.006	4	4.25 + 0.04	102.2	6.67
Cd	0.032 + 0.002	1	1.087 + 0.015	105.5	6.57	0.01 + 0.001	4	4.03 + 0.05	100.5	5.59
Co	0.553 ± 0.021	1	1.727 + 0.015	117.3	3.76	0.01 + 0.002	3	3.40 + 0.03	113	11.78
Cu	0.343 + 0.021	1	1.463 ± 0.032	112	6.06	0.21 + 0.003	3	3.13 + 0.04	97.3	2.79
Cr	0.283 + 0.011	1	1.337 + 0.015	105.3	4.1	0.39 + 0.001	3	3.59 + 0.02	106.7	13.48
Ni	0.147 + 0.015	1	1.160 + 0.030	101.7	10.4	0.25 + 0.001	3	3.45 + 0.03	106.7	8.92
Pb	0.207 + 0.008	1	1.209 + 0.002	100.2	3.94	0.59 + 0.002	3	3.70 + 0.04	103.7	4.44
Hg	0.042 + 0.001	1	1.223 + 0.011	118.1	2.38	0.06 + 0.001	4	4.21 + 0.07	103.7	5.41
Zn	0.142 + 0.007	1	1.217 + 0.090	107.5	4.62	0.17 + 0.001	3	3.69 + 0.03	117.3	9.75

A = concentration before spiking, B = amount spiked, and C = concentration after spiking.

**Table 3:** Heavy metal concentrations (mg·kg^−1^) in the soil from the study sites (mean ± SD, *n* = 15).

Site	As	Pb	Zn	Cd	Hg	Cu	Ni	Co	Cr
S1	5.58 ± 1.26^a^	42.34 ± 35.55^b^	62.37 ± 9.51^a^	1.10 ± 0.36^a^	2.04 ± 0.37^b^	10.03 ± 3.23^a^	6.98 + 2.33a	2.37 + 1.28^a^	10.05 + 1.37^a^
S2	5.82 ± 2.06^a^	43.7 ± 35.36^a^	60.67 ± 13.61^b^	1.23 ± 0.6^a^	2.34 ± 0.65^a^	8.57 ± 3.01^b^	8.43 + 5.24b	3.52 + 2.08^b^	9.52 + 4.1^b^
S3	3.04 ± 0.14^b^	12.75 ± 0.62^c^	35.89 ± 1.14^c^	0.32 ± 0.03^b^	1.38 ± 0.05^c^	3.71 ± 0.19^c^	1.81 + 0.18^c^	0.89 + 0.05^c^	3.39 + 0.29^c^
All S	5.17 ± 1.76	**30.9** ± 24.31	**56.39** ± 14.06	**1.0** ± 1.76	**1.96** ± 0.54	8.21 ± 3.47	6.53 ± 4.12	2.60 ± 1.99	8.50 ± 3.58
MPL	14	10	50	0.3	0.3	20	50	8	100

Mean values with the same superscript letters in a column are not significantly different (*p* > 0.05) from each other at *α* = 0.05. MPL = maximum permissible limit for agricultural soils according to [27, 28]. Bold values show that the four elements are much higher than MPL.

**Table 4:** Comparison of heavy metal concentrations (mg·kg^−1^) in soil reported from the nearby Ethiopian rift valley areas with the present study.

Study area	Mean concentrations in mg·kg^−1^ at sampling site(s)									Reference
	As	Pb	Zn	Cd	Hg	Cu	Ni	Co	Cr	
Hawassa vegetable farms	6.7	10.9	133.0	0.2	—	28.7	14.0	—	19.7	Samuel et al. [47]
	8.4	12.9	140.0	0.3	—	73.3	22.0	—	26.1	

Modjo vegetable farms	24.1	37.93	98.9	5.3	6.3	26.0	35.6	15.1	36.2	Gebeyehu and Bayissa [48]
	24.1	35.80	93.7	4.8	7.3	25.5	30.5	14.9	35.9	

Koka vegetable farms	20.9	37.30	97.8	4.4	6.1	19.8	35.0	13.5	48.1	Bayissa and Gebeyehu [49]
	27.7	43.60	108.3	6.0	6.7	24.0	40.3	15.9	49.2	
	29.8	47.20	126.8	6.0	7.7	26.3	42.5	18.9	49.9	
	31.4	48.60	138.9	6.4	8.2	28.7	50.7	21.7	60.7	

Ziway vegetable farms	3.0	12.75	35.9	0.3	1.4	3.7	1.8	0.9	3.4	This study
	5.6	42.34	60.7	1.1	2.0	8.6	7.0	2.4	9.5	
	5.8	43.70	62.4	1.2	2.3	10.1	8.4	3.5	10.1	

**Table 5:** Heavy metal concentrations (*μ*g·L^−1^) in water samples collected from the three sampling sites (*n* = 15; specific site for minimum and maximum values is indicated in bracket).

Heavy metals	Concentration (*μ*g·L^−1^) (mean ± SD)			RML (*μ*g·L^−1^)
	All sites (*n* = 15)	Min. value	Max. value	
As	80 ± 10	50 (S3)	110 (S1)	100
Pb	570 ± 30	520 (S3)	610 (S1)	5000
Zn	170 ± 30	130 (S3)	200 (S1)	2000
Cd	6 ± 3	3 (S3)	9 (S2)	10
Hg	90 ± 20	60 (S1)	110 (S2)	—
Cu	140 ± 30	20 (S3)	180 (S1)	200
Ni	170 ± 10	110 (S1)	180 (S2)	200
Co	10 ± 4	8 (S3)	20 (S2)	50
Cr	50 ± 10	40 (S1)	69 (S2)	100

RML = recommended maximum limit for irrigation water of vegetable farms by FAO [50].

**Table 6:** Single heavy metal soil contamination (CF) and overall soil contamination (*C*_*d*_) at different sampling sites, *n* = 15.

Site	CF									*C*_*d*_	PLI
	As	Pb	Zn	Cd	Hg	Cu	Ni	Co	Cr		
1	0.35	0.78	0.39	1.22	2.32	0.26	0.21	0.35	0.13	6.01	0.449
2	0.36	0.80	0.38	1.33	2.66	0.22	0.25	0.41	0.12	6.53	0.467
3	0.19	0.23	0.23	0.33	1.59	0.09	0.05	0.06	0.04	2.81	0.157

**Table 7:** Single element potential ecological risk factor (ER) and potential ecological risk index (ERI) at different sampling sites, *n* = 15.

Site	ER									ERI
	As	Pb	Zn	Cd	Hg	Cu	Ni	Co	Cr	
1	3.5	3.9	0.39	36.6	92.8	1.3	1.05	1.75	0.26	141.55
2	3.6	4.0	0.38	39.9	106.4	1.1	1.25	2.05	0.24	158.92
3	1.9	1.2	0.23	9.9	63.6	0.45	0.25	0.3	0.08	77.86

**Table 8:** Pearson's correlation matrix between heavy metal concentrations in soils from Ziway irrigated horticultural farms.

	As	Pb	Zn	Cd	Hg	Cu	Ni	Co	Cr
As	1								
Pb	0.25	1							
Zn	**0.92** ^*∗∗*^	0.24	1						
Cd	**0.94** ^*∗∗*^	0.55^*∗*^	**0.88** ^*∗∗*^	1					
Hg	**0.85** ^*∗∗*^	−0.13	0.**77**^*∗∗*^	**0.71** ^*∗∗*^	1				
Cu	0.07	**0.75** ^*∗∗*^	0.18	0.29	−0.17	1			
Ni	**0.96** ^*∗∗*^	0.34	**0.93** ^*∗∗*^	**0.96** ^*∗∗*^	**0.80** ^*∗∗*^	0.08	1		
Co	0.20	**0.99** ^*∗∗*^	0.19	0.51	−0.15	0.75^*∗∗*^	0.30	1	
Cr	**0.89** ^*∗∗*^	0.58^*∗*^	**0.88** ^*∗∗*^	**0.95** ^*∗∗*^	0.63^*∗*^	0.44	**0.91** ^*∗∗*^	0.54^*∗*^	1

^*∗∗*^Correlation is significant at the 0.01 level (2-tailed). ^*∗*^Correlation is significant at the 0.05 level (2-tailed). Bold values show there is significant strong positive relationship.

**Table 9:** Principal component loading of heavy metals.

Heavy metals	PC1	PC2
As	**0.984**	0.125
Pb	0.167	**0.949**
Zn	**0.913**	0.291
Cd	**0.947**	0.297
Hg	**0.924**	−0.099
Cu	0.012	**0.808**
Ni	**0.966**	0.220
Co	0.419	**0.856**
Cr	**0.868**	0.458
% variance	**62.1**	**18.9**

Bold values show strong significant positive relationship.

## Data Availability

All data used to support the study findings are included within the article.
